# Attaining threshold antibody cytotoxicity for selective tumor cell destruction: an opinion article

**DOI:** 10.18632/oncotarget.26271

**Published:** 2018-11-06

**Authors:** Victor I. Seledtsov, Galina V. Seledtsova

**Affiliations:** ^1^ lmmanuel Kant Baltic Federal University, Kaliningrad, Russia; ^2^ Institute for Fundamental and Clinical Immunology, Novosibirsk, Russia

**Keywords:** therapeutic antibody, antibody-mediated cytotoxicity, immune complex, tumor destruction

## Abstract

We propose a novel immunotherapeutic paradigm that justifies application of several antibodies to various membrane-associated antigens to achieve a critical threshold density of immune complexes on the surface of cancer cells sufficient for triggering downstream cytolytic pathways. Indeed, some cancer-associated antigens (such as cancer/testis antigens) were found to be expressed on many cancer (but not normal) cells, with their baseline membrane expression levels being originally quite low for some of them, or even further down-regulated due to immune-driven cell selection. To achieve the mandatory threshold density of membrane-associated immune complexes on malignant cells, the concept stipulates combined application of antibodies specific for a cancer-associated antigen along with antibodies against an antigen expressed not only on tumor, but also on normal cells. In the proposed scenario it is of vital importance that the latter antibodies should be applied in suboptimal dosage to exclude the destruction of normal cells devoid of a cancer-associated antigen. Malignant cells often co-express antigens not present concurrently on normal cells at high levels. In such cases, suboptimal dosages of antibodies specific for those antigens could also be applied to achieve cumulative effect leading to selective destruction of tumour cells. Hence, the described immunotherapeutic technology could be used metaphorically speaking as a kind of ‘immunological knife’, which is capable of highly selective destruction of cancer cells without destroying normal cells.

## INTRODUCTION

Antibody-mediated cytotoxicity is based on four innate effects or mechanisms: complement-dependent cytotoxicity (СDC), antibody-dependent cell-mediated cytotoxicity (ADCC), antibody-dependent cellular phagocytosis (ADCP), and complement-dependent cellular cytotoxicity (CDCC). CDC is activated by IgM or IgG binding to the cell surface, thus triggering a cascade of more than 30 proteins culminating in the formation of the membrane-attack complex and subsequent cell destruction. ADCC and ADCP occur when effector cells (granulocytes, macrophages, and NK-cells) bind to target cells coated with antibodies (Abs) which initiates a downstream cytodestructive process. CDCC requires prior activation of complement and deposition of C1q, C3b, iC3b or C4b complement components on the target cell. In this respect, Abs that facilitate deposition and activation of complement components on the cell surface are of primary importance in triggering complement-dependent cell-mediated cytolysis [1–3].

The overall Ab-mediated cytotoxic effect is believed to be accounted for by a ‘threshold’ phenomenon that necessitates for the membrane-associated immune complexes to reach a certain threshold density in order to elicit downstream cytolytic cascades. The ‘threshold’ phenomenon was convincingly demonstrated in experiments with both polyclonal Abs against various Ags expressed on the cell surface [4–8] and monoclonal Abs that recognised only appropriate membrane-associated Ags [9–12]. The biological significance of the ‘threshold’ phenomenon has been postulated as providing a mechanism of damping excessive cytotoxicity potentially resulting from miniscule antigenic challenges harmless for the organism. Density of the immune complexes present on the cell membrane is determined by such factors, as expression levels of membrane-associated Ags, concentrations of the appropriate Ag-specific Abs present in the microenvironment, and affinity of Ag-Ab interactions, which controls stability of immune complexes formed on the cell surface [13]. Taken together, Ab-mediated cytotoxicity occurs when a number of high-affinity Ag-specific Abs effectively interact with appropriate Ags abundantly expressed on the cell surface. On the contrary, cytolytic process is not triggered when: (i) high (optimal) concentrations of Ag-specific Abs are available in the microenvironment as combined with low (suboptimal) expression of appropriate membrane-associated Аgs, and (ii) high expression levels of membrane-associated Аgs co-exist with low (suboptimal) concentrations of Abs of the appropriate specificity. In addition, it is the instability of the immune complexes formed on the cell membrane that can also provide a negative regulatory mechanism with respect to launching downstream cytolytic cascade reactivity.

## IMMUNOTHERAPEUTIC APPLICATION OF SUBOPTIMAL CYTOTOXIC ANTIBODY DOSAGES FOR TREATMENT OF CANCER PATIENTS

The main problem of anti-tumor systemic chemotherapy consists in the absence of selectivity of the cytotoxic activity of officinal anticancer drugs, which exert their effects not only on malignant, but also on normal cells. The development of chemotherapeutic drugs with targeted cytotoxic activity is unlikely in the foreseeable future because of the key biochemical pathways that are similar in tumor and normal cells. Nevertheless, tumor cells can be identified by quantitative and qualitative differences in potentially immunogenic molecules expressed on the cell surface. The current paradigm holds that antitumor immune mechanisms are capable of destructing tumor cells without destroying normal cells, and that the effective immunоtherapy could dramatically improve prognosis and clinical disease outcome [14].

All tumor-associated Ags can be conditionally divided into two subgroups. The first subgroup includes Ags expressed exclusively or preferentially on malignant cells, with the second subgroup shared between both cancer and normal somatic cells. Cancer/testis Ags (CTAs) and oncofetal Ags are encoded by awakened ‘silent’ genes. In adult organisms, CTAs are normally expressed exclusively in testes and, consequently, can be conditionally considered as tumor-specific Ags [14]. Although tumor-specific Ags are sufficiently immunogenic to induce tumour-destructive immune reactivity, cells with lower/absent expression of such Ags gain selective preferences during tumorigenic process, due to host antitumor immunoreactivity. Conceivably, active immunotherapeutic protocols designed to up-regulate anti-tumor immune reactivity in cancer patients could also exert selective pressure and facilitate growth of tumor cells characterised by low expression levels of immunogenic Ags and consequently by Ab-cell interactions occurring below the cytotoxic threshold [3].Evidently, the probability of achieving the required threshold cytolytic density of membrane-associated immune complexes would increase upon wider involvement of more membrane Ags into immune responses. This is the reason why polyantigenic anti-cancer vaccines are more effective in generating robust anti-tumor clinical effects, as compared to their monoantigenic counterparts [3]. However, in reality cancer afflicts predominantly elderly patients whose immune system was deteriorated by age-related changes. In addition, a malignant tumor itself exerts a negative effect on the host‘s immunity. The above-mentioned facts drastically restrict therapeutic potential of any active anti-cancer immunotherapeutic modality and urge paying more attention to passive immunotherapy protocols.

**Figure 1:**
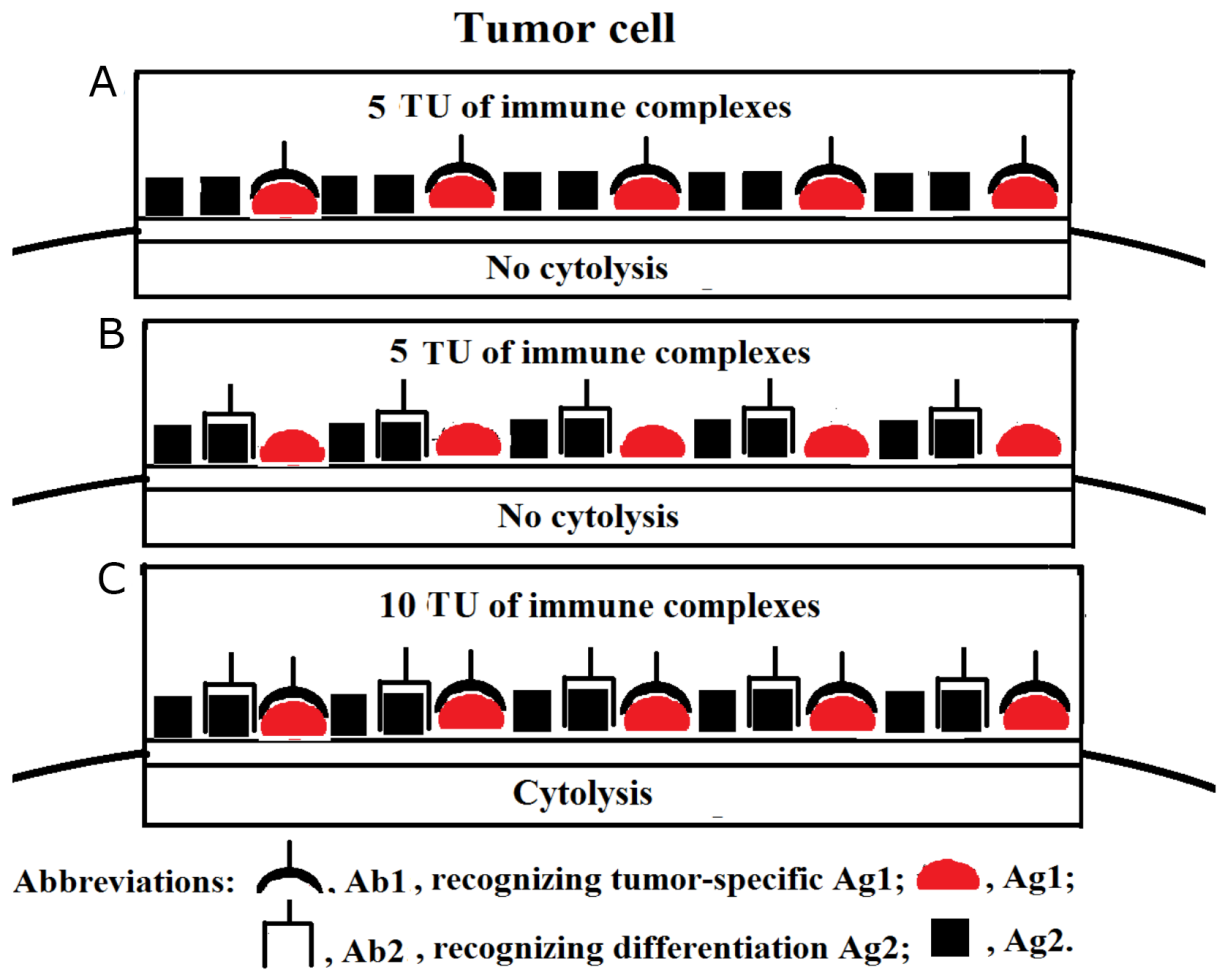
**(A, B, C)** Tumor cell cytolysis ensued as a result of cooperative activity of tumor-specific Ab and Ab against an Ag expressed not only on tumor, but also on normal cells. This theoretical scenario assumes that a critical threshold density of membrane-associated immune complexes constitutes 10 tentative units (TU) necessary and sufficient to cause cell lysis. Here, the optimal dose of tumor Ag1-specific Ab1 is sufficient to achieve density of immune complexes of just 5 TU (i.e. below the threshold level) on the surface of a tumor cell due to low level of Ag1 expression (A). Suboptimal concentration of Ab2 against an Ag2 expressed on both tumor and normal cells also fails to trigger tumor cell cytolysis due to Ab2 insufficiency present in cell microenvironment (B). Cooperative activity of both Ab1 and Ab2 facilitates the attainment of the required threshold levels for the membrane-associated immune complexes (10 TU), thus, triggering tumor cell lysis (C).

In the last decade, we witnessed active and serious growth of the immunotherapeutic market and in particular in its segment of low-immunogenic humanised and fully human Abs. Therapeutic Abs have become a backbone of routine treatment strategies for a variety of diseases, including malignancies. Modern bioengineering technologies allow for manufacturing low-immunogenic cytotoxic IgG Abs of nearly any specificity, and several anti-cancer Abs-based modalities (such as Rituximab, Trastuzumab, Cetuximab, and Panitumumab) have already been introduced into clinical practise [2, 15]. We believe that selective destruction of cancer cells can be achieved by using more than just one particular Ab preparation specific for a particular membrane-associated Ag expressed on cancer cells. Those Abs can be applied both in optimal and suboptimal dosages, and their net overall effect would add up to allow the immune complexes to reach a threshold cytolytic density only on those cancer cells that are recognised by all Abs included in the immunotherapeutic Ab formulation. Normal cells that have been recognised by some, but not all, Abs will remain alive because density of the membrane-associated immune complexes would be below critical cytotoxic threshold level. For instance, we postulate that in order to accomplish selective tumor cell destruction, cancer-specific Ab could be used in optimal concentration (if a particular cancer-specific Ag is expressed at a relatively low level) supplemented with suboptimal (subthreshold) dose of Ab to an Ag expressed not only in cancer, but also in normal tissues. Figure 1 illustrates a notion that although a single Ab was not able to induce cell lysis, the application of two Abs ensured recognition of two different molecular membrane targets and allowed to reach threshold density of membrane-associated immune complexes required for cell lysis. On the practical clinical level, we envisage that patients with squamous cell carcinoma could be potentially treated with any tumour-specific Ab (recognizing, for example, any cancer/testis Ag) in combination with Ab specific for epidermal growth factor receptor (HER1 in humans) expressed both on tumor and normal cells. On a similar note, patients with sarcoma could be treated with tumour-specific therapeutic Ab along with Ab against fibroblast growth factor receptors, with analogous strategy applied for other malignancies. Noteworthy is that local inflammation in tumor environment could improve the availability of malignant cells for the action of therapeutic Abs.

As was stressed above, not all tumor cells express significant levels of tumour-specific Ags, and such cells often demonstrate co-expression of Ags (including oncogene products) which are not present concurrently on normal cells at high levels. Thus, abnormally high co-expression levels of β-catenin and E-cadherin has been demonstrated on gastric carcinoma cells [16], while CD44V6 and β-catenin are co-expressed in osteosarcoma [17]. Such an abnormal Ag expression is explained by genetic instability of tumor cells and high growth activity of some tumor cell clones. In such circumstances, a combined application of suboptimal doses of Abs against Ags co-expressed on the cell surface could be indicated with a view to obtain cumulative Ab effects for selective destruction of tumor (but not normal) cells. Our immunotherapeutic concept could be extended to include more than two individual Ab-based therapeutics. Theoretically, the increase in the number of target Ags should be paralleled by the decrease in dosages of all relevant Ag-specific Abs that contribute to the selective net cytolytic effect in the system, which, in turn, would reduce risks of developing side effects associated with the application of a particular Ab-based drug.

We feel that it is important to stress that low concentrations of autoantibodies against differentiation membrane Ags are frequently found in serum of healthy subjects [18], and those levels could be further up-regulated in response to infections [19]. According to our concept, the role for such auto-Abs could consist in raising sensitivity of the immune system to effector recognition not only of cancer cells, but also infected cells. However, if cumulative auto-Ab levels to various membrane-associated molecules could reach threshold density of immune complexes on normal cells, such scenario could lead to autoimmune diseases. Therefore, the threshold phenomenon of Ab-mediated cytotoxicity could potentially fuel the development of autoimmune diseases.

There have been several technological platforms developed recently that allow for determination of threshold (and sub-threshold) Ab doses in various experimental and translational settings [20–22], which could be applied for establishing particular dosages of Ab-based drugs in the context of the proposed paradigm. We hypothesise that complex Ab preparation would be characterised by a certain therapeutic range, as is the case with other drugs, with doses of individual Abs having been preliminary established in order to ensure maximal clinical effects (i.e. destruction of tumor cells) and minimal side effects (i.e. preservation of normal cells). Clearly, any Ab formulation designed for selective elimination of tumor cells should be standardised and applied in controlled clinical settings in a specialised facility taking into account weight and body surface area of the patient. Certainly, in cases of individual variations in target marker levels, Ab concentrations could be optimised accordingly on a patient-to-patient basis. In addition, in accordance with procedures applied for other preparations, individual dosing schedules for an Ab drug would be adjusted with regard to disease severity and disease state.

It is important to note that mechanisms underlying Ab-mediated cytotoxicity are under tight regulation by the immune system with many membrane-associated and soluble immune factors involved. From the practical clinical perspective, it is important that immune cell disfunctions, complement depletion, as well as IgG-containing immune complexes present in patients’ serum, could cause reduction in Ab-mediated cytotoxicity [2]. Clearly, this necessitates passive Ab-based immunotherapy regiments to be highly personalised to include a comprehensive set of measures (such as transfusion of donor leukocytes and/or of fresh complement-sufficient plasma preparations) in order to maximize clinical effectiveness. Our concept suggests that therapeutic Abs conjugated with artificial cytotoxic molecules could be also applied for selective tumor destruction. The main requirement for such molecules should be the existence of an achievable quantitative threshold for triggering their cytotoxic action. Hence, the described immunotherapeutic technology could be used metaphorically speaking as a kind of ‘immunological knife’, which could potentially achieve highly selective destruction of cancer cells without destroying normal cells.

## Abbreviations

Ab: antibody
Ag: antigen
ADCC: antibody-dependent cell-mediated cytotoxicity
ADCP: antibody-dependent cellular phagocytosis
СDC: complement-dependent cytotoxicity
CDCC: complement-dependent cellular cytotoxicity
CTA: cancer/testis antigen
